# PROTAC-mediated multi-target protein degradation in Alzheimer's disease: mechanistic insights, therapeutic applications, and translational challenges

**DOI:** 10.1039/d5md00963d

**Published:** 2026-02-20

**Authors:** Bin Wang, Yunan Li, Tingting Yao, Xinai Shen, Huan Li, Wei Jiang, Xinuo Li, Zheying Zhu

**Affiliations:** a State Key Laboratory of Natural Medicines, China Pharmaceutical University 639 Longmian Avenue Nanjing 21198 China xinuo.li@cpu.edu.cn +86 15298351729; b School of Pharmacy, The University of Nottingham Nottingham NG7 2RD UK Zheying.Zhu@nottingham.ac.uk

## Abstract

Alzheimer's disease (AD) is a progressive neurodegenerative disorder characterized by multiple interacting pathological mechanisms. Among these, the core driving factor is the accumulation of abnormally folded, neurotoxic proteins. Current therapeutic options, particularly immunotherapies, remain limited due to their reliance on immune-mediated clearance, the need for high-dose administration, and their inability to access intracellular targets, making them less patient-friendly and therapeutically efficient. This is precisely where proteolysis-targeting chimera (PROTAC) technology offers a transformative advantage. By harnessing the ubiquitin–proteasome system (UPS), PROTACs can selectively recruit degradation machinery to target proteins, thereby facilitating their ubiquitination and subsequent clearance. This mechanism allows PROTACs to efficiently eliminate pathogenic proteins while overcoming several inherent limitations of both traditional occupancy-based inhibitors and antibody-based therapies. In this review, we compare currently available AD therapies to highlight the advantages and mechanistic rationale underlying the promise of PROTAC technology in this field. We further discuss the molecular principles of PROTAC-mediated protein degradation and its emerging applications in tackling protein aggregation pathologies, focusing on both established and novel therapeutic targets. By drawing insights from existing PROTAC studies, we aim to inform future design strategies that reduce off-target effects, accelerate candidate optimization, and ultimately contribute to the development of a new generation of AD therapeutics.

## Abstract 2

Clearing pathological proteins in Alzheimer's disease *via* PROTAC-induced degradation.

## Graphical abstract

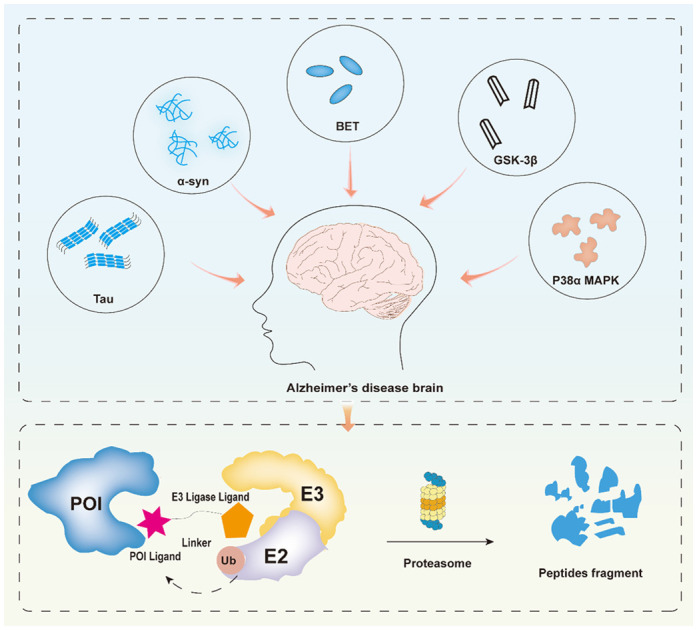

## 1. Introduction

Alzheimer's disease (AD) is the most common age-related neurodegenerative dementia, characterized by oxidative stress, synaptic dysfunction, and progressive cognitive and memory decline. Its complex pathogenesis, coupled with the lack of effective therapeutic interventions, makes it a major global health challenge.^1–3^ It is estimated that by 2060, approximately 13.8 million people will be affected by AD, with more than 110 000 AD-related deaths reported in 2021, imposing substantial burdens on both society and families.^4,5^ As the most prominent pathological hallmarks of AD, the formation and accumulation of amyloid plaques and neurofibrillary tangles (NFTs) are widely recognized as central events driving disease onset and progression. Amyloid plaques arise primarily from the aberrant aggregation of β-amyloid (Aβ) peptides, which are generated through the pathological cleavage of amyloid precursor protein (APP). Due to inherent structural instability, the resulting Aβ peptides undergo conformational rearrangements and progressive aggregation, transitioning from soluble oligomers—recognized as the most neurotoxic species—to insoluble fibrils, and ultimately forming amyloid plaques; this process is widely regarded as a primary underlying driver of AD pathogenesis.^6^ Concurrent with amyloid plaque formation is the development of NFTs, which consist of abnormal filamentous aggregates of hyperphosphorylated tau proteins (p-Tau) within neurons. Notably, tau hyperphosphorylation drives the formation of these tangles, ultimately compromising neuronal structure and function, a phenomenon acknowledged as another cardinal pathological feature of AD.^7,8^

With advances in research, Aβ aggregation and tau hyperphosphorylation are now being recognized as interconnected events within a complex network, with neuroinflammation serving as a central regulatory hub.^9^ Evidence of neuroinflammation has been observed through biofluid biomarkers in cerebrospinal fluid (CSF) and molecular imaging techniques such as positron emission tomography (PET) in patients with AD, revealing excessive activation of microglial cells in the brain.^10^ It has been found that not only Aβ burden and NFTs contribute to neuroinflammation but also various cellular and molecular pathways play crucial roles,^11^ for instance, the mTOR pathway mediated by GSK-3β, which activates microglia; epigenetic mechanisms such as histone acetylation, which modulate microglial dynamics; and the involvement of other misfolded proteins from other neurodegenerative diseases, such as α-synuclein, whose aggregates can be internalized, propagated, and induce pathological activation within brain immune cells.^12^

The classical US Food and Drug Administration (FDA)-approved treatments for AD are primarily small-molecule inhibitors targeting acetylcholinesterase (AChE) or the *N*-methyl-d-aspartate (NMDA) receptor, which help improve overall neuronal function.^13,14^ The later FDA-approved therapies, beginning with aducanumab in 2021,^15^ has shifted toward passive antibody-based anti-amyloid treatments, designed to target various stages of Aβ pathology. Among the most recent approvals, lecanemab and donanemab specifically target the Aβ protofibril and senile plaque stages, respectively. However, current immunotherapies face several challenges due to their large molecular size, low brain exposure, dependence on immune-mediated clearance, and the need for frequent infusions at high doses.^16^ These limitations not only reduce treatment efficacy but also raise concerns regarding adverse effects, such as cerebral amyloid angiopathy, making these therapies less patient-friendly.

Therefore, the development of new treatments that achieve similar protein degradation effects but rely less on the immune system and require lower drug dosages may offer a promising alternative to immunotherapy. This consideration has inspired the design of novel strategies for targeted protein degradation, among which the proteolysis targeting chimera (PROTAC) technology stands out as a promising one. PROTACs function by harnessing the ubiquitin–proteasome system (UPS) to selectively degrade ubiquitinated proteins, operating on the basis of a catalytic-like cycle that allows a single PROTAC molecule to induce the degradation of multiple target proteins.

In this review, by comparing currently available therapies for AD, we aim to highlight the advantages and rationale behind the promising potential of PROTAC technology in this field. Furthermore, we seek to elucidate the mechanisms of PROTAC-mediated protein degradation and explore its applications in addressing protein-aggregating pathology, with a particular focus on both well-studied and emerging therapeutic targets. We hope that by learning from the currently available PROTACs, future research can be guided toward more rational design strategies, minimizing unwanted effects and expediting the characterization of candidates, ultimately paving the way for a new generation of AD therapeutics.

## 2. Current status of AD drug treatment

### 2.1 First generation of FDA-approved drugs

The FDA has sanctioned a limited number of conventional medications for managing AD, notably donepezil, rivastigmine, galantamine, and memantine.^17,18^ The initial three are inhibitors of AChE, while memantine functions as an antagonist of the NMDA receptor (Table 1). Despite their widespread utilization, ongoing research is dedicated to refining their dosing, formulation, route of delivery, and amalgamated therapies to mitigate adverse reactions and enhance patient adherence.^19^ Notably, the FDA endorsed the use of donepezil transdermal patches (Adlarity) in 2022 for addressing mild, moderate, and severe AD.^20^ Studies propose that conjoining donepezil and/or galantamine with neuroprotective agents, metal chelators, or antioxidants could notably enhance the effectiveness, tolerability, and safety of AD cholinergic pharmacotherapy.^21,22^ Nevertheless, these medications primarily ameliorate symptoms by modulating neurotransmitter levels and are incapable of modifying the disease's progression.

**Table 1:** First generation of FDA-approved drugs: small-molecule inhibitors

Drug	Structures	Mechanism
Donepezil	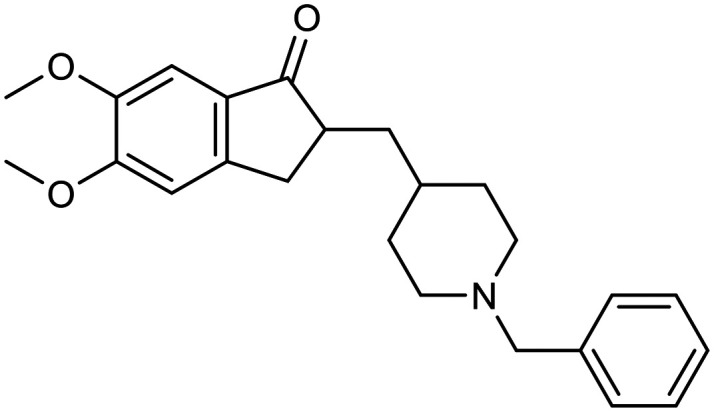	AChE inhibitors
Rivastigmine	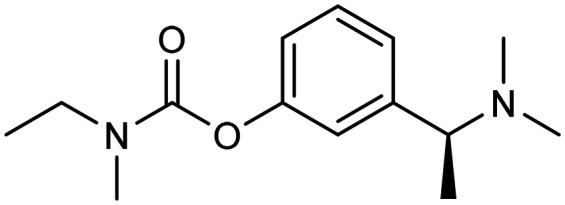	
Galantamine	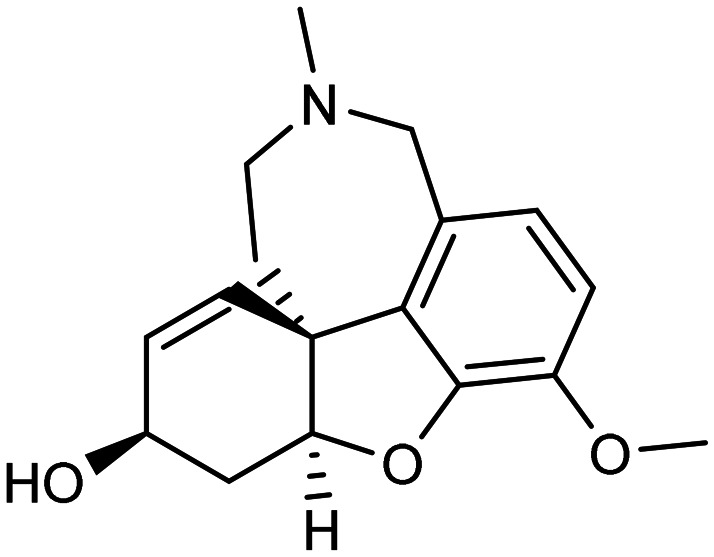	
Memantine	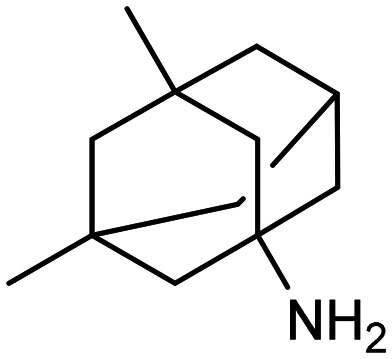	NMDA antagonist

### 2.2 Second generation of FDA-approved drugs (2021 onwards)

Notable advancements have been achieved in disease-modifying therapies (DMTs), especially monoclonal antibodies directed against pathological proteins implicated in AD. Aducanumab, approved in 2021, specifically interacts with the 3rd to 7th amino acids of the Aβ peptide, with specificity for amyloid species ranging from oligomeric to fibrillar forms.^23^ However, the clinical effectiveness of aducanumab remains a topic of debate.^24^ In 2023, FDA granted full approval to lecanemab, an antibody that targets the E22G Aβ epitope with a notably higher affinity for soluble Aβ aggregates, especially in the form of protofibrils, compared to aducanumab, effectively preventing their aggregation into toxic oligomers and fibrillar deposits.^25^ However, treatment with these antibodies may be linked to adverse events like amyloid-related imaging abnormalities (ARIA), and prolonged use could result in severe adverse reactions.^26,27^

In 2024, another monoclonal antibody was approved for early-stage AD patients carrying fewer than two copies of the apolipoprotein E4 (ApoE4) allele.^28^ This antibody specifically targets the cyclized pyroglutamate of the third amino acid in Aβ, in which both aspartate-1 and alanine-2 are absent (known as N3pG Aβ).^29^ This peptide variant is found exclusively in amyloid plaques, which are largely insoluble. Although amyloid plaques are typically associated with the later AD pathogenesis, administering the antibody to early-stage patients, whose brains are not yet dominated by plaques,^30^ may limit the therapeutic benefit.

### 2.3 Limitations of current immunotherapies

Beyond FDA-approved immunotherapies, numerous DMTs are under development that focus on other pathological proteins of interest, including tau pathways.^31,32^ For instance, the anti-tau monoclonal antibody E2814 targets the microtubule-binding domain of tau to impede protein aggregation and “seed” amplification.^33^ According to phase I data, optimal target engagement was achieved at a single intravenous (IV) dose of 60 mg kg^–1^ in healthy subjects, although the effective concentration in patients may vary.^34^ Currently, four clinical trials are underway: a phase I/II trial on autosomal dominant AD patients (NCT04971733), one phase II trial and two phase II/III trials investigating the combination of lecanemab and E2814 for early-onset AD (NCT06602258, NCT01760005, NCT05269394).

Another amyloid-directed immunotherapy, PMN310, is designed to selectively target amyloid oligomers while minimizing ARIA commonly associated with Aβ immunotherapies. Its ongoing phase Ib trial (NCT06750432) is recruiting early AD patients to evaluate dose levels ranging from 350 mg to 1400 mg. Gantenerumab, a subcutaneously administered anti-Aβ antibody that targets soluble oligomers, fibrils, and plaques, showed promise in preclinical studies. However, two phase III trials (NCT03444870, NCT03443973) were terminated due to insufficient clinical efficacy in delaying cognitive decline among patients with prodromal to mild AD, despite high-dose treatment (510 mg every two weeks for up to 116 weeks).^35,36^

Taken together, current clinical trials suggest that antibody-based therapies exhibit limited translational potential from preclinical studies to clinical practice, with limited efficacy observed in moderate to severe cases. To better understand these limitations, it is essential to examine the mechanistic basis of immunotherapy. As current market-approved treatments for AD primarily rely on passive immunotherapy, this section will focus on that approach, while active immunotherapy, which induces endogenous antibody production, will not be further discussed.

Passive immunotherapy (Table 2), including all above-mentioned monoclonal antibodies, works by antibody-guided labelling of the antigen, in this case the drug target, followed by clearance from effector cells of the immune system, triggering the process of antibody-dependent cellular cytotoxicity (ADCC) or antibody-dependent cellular phagocytosis (ADCP) to eliminate the drug target.^37,38^ Therefore, a successful degradation of target in the brain using immunotherapy depends mainly on target specificity, binding stability, and immune effectiveness. Because effector cells recognize the Fc region of antibodies, the antibody–antigen complex must remain stable long enough for the subsequent clearance to occur. Moreover, given that each antibody can bind to one antigen at once, a high molar quantity of antibody is required to label the abundant pathological proteins such as Aβ. This explains the need for frequent and prolonged infusions, as seen with lecanemab in clinical settings.^39^

**Table 2:** Passive immunotherapy *vs.* PROTAC

	Passive immunotherapy	PROTAC
Effect upon immediate drug–target combination	Neutralization of target antigen	Induction of ubiquitination
Structural organization	Integrated single macromolecule	Bifunctional molecule consisting of two ligands connected by a linker
Average molecular weight	∼150 kDa (IgG)	∼0.8–1.2 kDa
Drug–target interaction	Many: 1	1: many (catalytic)
Site of action	Extracellular	Intracellular
		Extracellular^40,41^
Downstream effect	Antibody-dependent cellular cytotoxicity (ADCC), antibody-dependent cellular phagocytosis (ADCP), and other immune-mediated clearance pathways	Targeted protein degradation *via* the ubiquitin–proteasome system (UPS)
Reliance on immune system	High	Low
Blood–brain barrier (BBB) permeability	Poor	Moderate to good (depending on molecular design)

In addition, the large molecular weight typical of biologics restricts blood–brain barrier (BBB) penetration following IV administration, necessitating higher systemic doses. Although strategies such as antibody–drug conjugate (ADC)-like technology^42^ or engineered Fab or Fc modifications^43,44^ have been developed to enhance BBB transport by introducing BBB receptor ligands, these modifications increase structural complexity, posing obstacles for Fc-mediated clearance. Furthermore, the presence of such large exogenous complexes can elicit high immunogenicity that further compromises therapeutic efficacy.

Finally, the nature of immunotherapy, which relies on extracellular targeting and the recruitment of effector cells, presents additional limitations. Increasing evidence suggests that intracellular targets play crucial roles in AD pathogenesis,^45,46^ making anti-extracellular approaches alone less promising to all disease stages. Moreover, AD patients often exhibit an impaired immune response within the central nervous system (CNS). For instance, dysfunction of microglia^47–49^—the primary effector cells in the brain—may further compromise the efficacy of antibody-based therapies, contributing to variability in treatment outcomes across individuals.

### 2.4 Next generation of AD treatment

The next generation of therapeutic strategies in AD should focus on achieving maximal efficacy with minimal drug dosage and engaging with CNS-penetrant design, thereby reducing the risk of excessive immune activation and improving BBB penetration. In addition, therapies capable of accessing intracellular targets are urgently needed to intervene in the early molecular events underlying AD pathogenesis.

To address these challenges, a novel approach of protein degradation has emerged—PROTACs (Table 2). Unlike traditional small-molecule inhibitors, which depend on sustained high-affinity binding to suppress target activity, PROTACs function *via* an event-driven mechanism to induce selective protein degradation, offering the advantages of lower molecular weight and independence from immune-mediated clearance typical of antibodies. Acting catalytically, each PROTAC molecule can trigger the destruction of multiple copies of its target protein, enabling effective action at lower doses. Such a mechanism not only reduces the frequency of administration but also makes PROTACs particularly well suited for the long-term treatment of chronic, age-related conditions like AD.

## 3. PROTACs as the emerging technology for protein degradation

PROTAC technology, first reported in 2001, has since gained widespread application in drug discovery owing to its unique ability to eliminate rather than merely inhibit proteins.^50,51^ PROTACs are bifunctional molecules comprising a target protein ligand (POI), an E3 ligase ligand, and a linker connecting them. By simultaneously binding the POI and the E3 ligase, PROTACs exploit the cell's UPS to degrade the targeted proteins.^52,53^

**Fig. 1:**
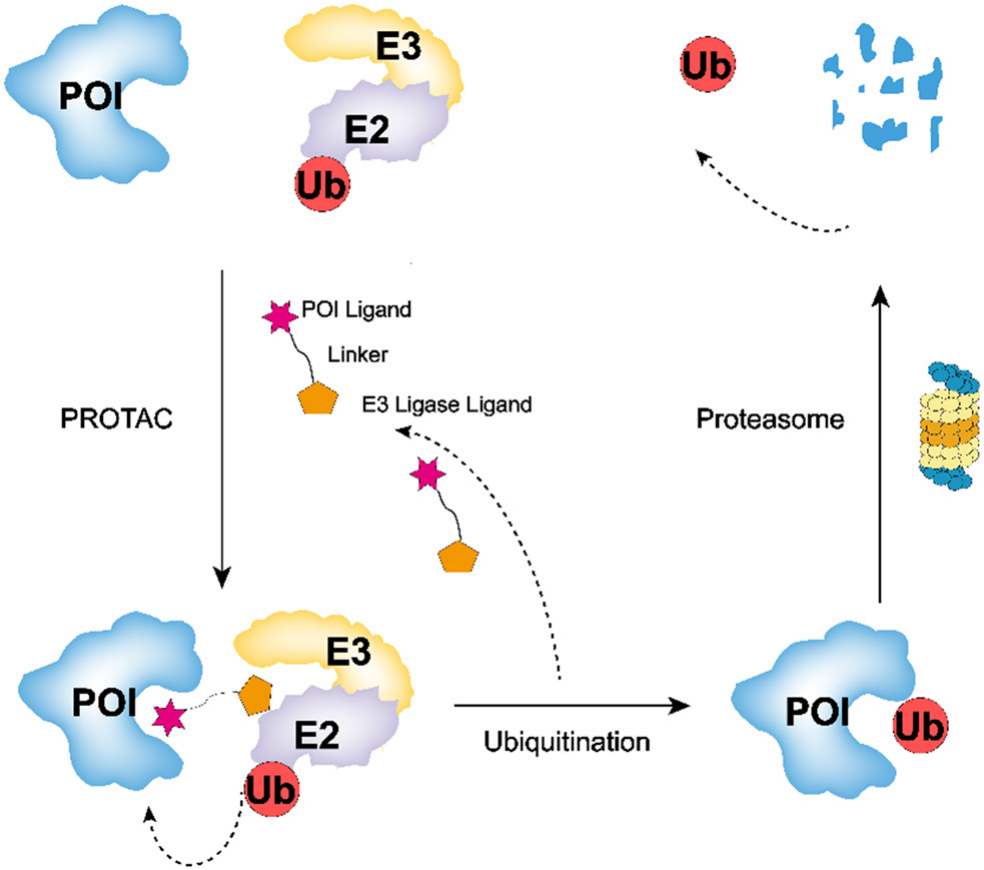
Mechanism of proteolysis-targeting chimera.

This process is mediated by a cascade of events (Fig. 1), in which ubiquitin (Ub) is first activated by E1 ubiquitin-activating enzymes in an ATP-dependent reaction and subsequently transferred to E2 ubiquitin-conjugating enzymes *via* transesterification, forming the E2–Ub complex. Concurrently, PROTAC molecules recruit the protein of POI to an E3 ubiquitin ligase, juxtaposing the substrate and ligase. The E3 ligase then catalyzes the transfer of Ub from the E2–Ub complex to lysine residues on the POI, initiating ubiquitination.^54–56^ The ubiquitinated target protein is then recognized and degraded by the 26S proteasome (Fig. 1), completing the targeted protein degradation process.^54,57^

PROTACs surpass traditional small-molecule inhibitors in several key aspects. Unlike conventional drugs that require strong and sustained target engagement, PROTACs require only weak binding to initiate target protein degradation, thereby enabling the targeting of previously “undruggable” targets.^58,59^ In addition, due to their catalytic mechanism—where the PROTAC facilitates target ubiquitination and is subsequently released from the drug–target complex—PROTACs can achieve sustained protein degradation at sub-stoichiometric concentrations, enhancing therapeutic efficacy while reducing the risk of off-target toxicity.^60^

The PROTAC technology has introduced a novel therapeutic approach for traditionally challenging targets. Over the course of almost twenty years, this technology has transitioned swiftly from fundamental investigation to clinical validation. By February 2026, three PROTAC drugs globally have progressed to phase III clinical trials, with more than 30 potential drugs in various clinical phases across a range of areas including hematological malignancies, solid tumours, and autoimmune disorders.^61^ ARV-471 (vepdegestrant), which targets estrogen receptors, is the first PROTAC drug to enter phase III clinical trials and has submitted a marketing application.^62^ It is expected to be the inaugural product approved for marketing that employs this technology, marking a significant transition of PROTAC from concept to clinical application. Nevertheless, research on PROTACs designed to degrade pathogenic proteins in AD remains relatively sparse. Currently, no related molecules have advanced to clinical research. Consequently, to accelerate the development of PROTAC drugs for AD treatment, it is crucial to further investigate the pathological mechanisms underlying AD and to systematically summarize the design experiences of existing PROTAC molecules. This strategy will promote the emergence of more brain-targeted degraders with therapeutic potential.

## 4. Development of PROTAC in Alzheimer's disease

The fundamental principle of the drug strategy remains the same as in anti-tumour research: to achieve therapeutic effects through the selective degradation of disease-driving proteins. Here, we introduce several PROTACs that have been developed to eliminate misfolded or aberrant proteins associated with neuronal toxicity, which represents the major pathogenic hallmark of neurodegenerative diseases, especially AD. Specifically, the dysregulated proteins include tau protein, glycogen synthase kinase-3β (GSK-3β), bromodomain and extraterminal (BET) proteins, α-synuclein and P38α MAPK, all of which contribute to the pathogenesis of neurodegenerative disorders. We will also discuss additional potential targets for PROTAC-based interventions that have not yet been explored experimentally, including aberrant proteins or signalling pathways of ApoE4, Fyn kinase, hypoxia/HIF1α signaling, PI3K/AKT/mTOR pathway and HDAC11.

### 4.1 PROTACs targeting tau proteins

One of the pathological hallmarks of neurodegenerative diseases, particularly in AD, is the abnormal accumulation and aggregation of tau proteins. These cytotoxic proteins play a crucial role in maintaining neuronal microtubule stability and function while facilitating nutrient transport and neurotransmission.^63^ In AD, p-Tau promotes the formation of NFTs, which compromise microtubule stability and subsequently disrupt axonal transport and synaptic communication.^64^ This cascade ultimately contributes to neuronal dysfunction and death. Consequently, p-Tau has emerged as a prominent therapeutic target in AD.^65^

Since the first demonstration of specific knockdown of endogenous tau protein using PROTAC by Li, Yan-Mei *et al.* in 2016,^66^ extensive studies have reported tau-targeting PROTACs incorporating various E3 ligase ligands, including von Hippel–Lindau (VHL) and cereblon (CRBN).

Among the E3 ubiquitin ligases with VHL as ligase-binding motif, in 2019, R. B. Kargbo *et al.* established a series of PROTACs targeting tau protein, with one promising compound (Table 3, PROTAC 1) extensively validated through multiple assays.^67^ Subsequently, in 2021, Wang's team developed a structurally distinct, non-peptide PROTAC (Table 3, PROTAC 2) that demonstrated markedly faster absorption, exhibiting a substantially shorter *T*_max_ in both plasma (0.1 h *vs.* 1 h) and brain (0.1 h *vs.* 0.5 h) compared to PROTAC1. With evaluation from *in vivo* and *in vitro* studies, PROTAC2 showed potent tau clearance, with an IC_50_ of 7.85 nM in HEK293-hTau cells. *In vivo* studies demonstrated significant tau reduction in wild-type, hTau transgenic, and 3xTg-AD mice following single and repeated subcutaneous injections, surpassing the efficacy of conventional intracerebroventricular delivery. Behavioural assessments and synaptic plasticity measurements further revealed improved cognitive function. However, the compound exhibited a dose-dependent “hook effect”, a false-negative phenomenon observed in PROTACs, where excessively high concentrations of the compound hinder the formation of the productive ternary complex, thereby reducing degradation efficiency. To enable clinical advancement, the drug may need to be optimized to improve oral bioavailability, given its suboptimal brain exposure. Furthermore, its long-term neurological safety and efficacy must be further established.^68^

**Table 3:** Tau-targeting PROTACs

PROTAC	POI	E3 ligase	Structure	Efficacy validation model	Ref.
1	Tau	VHL	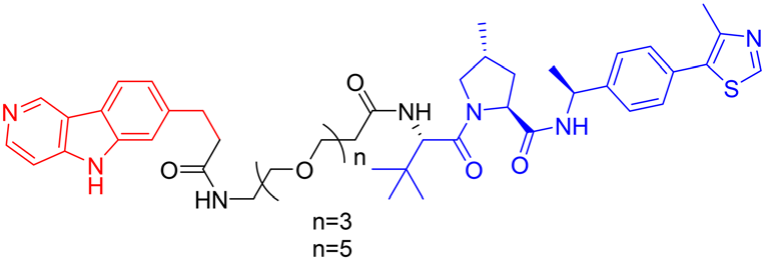	*In vitro* (tau-overexpressing human cells)	67
2			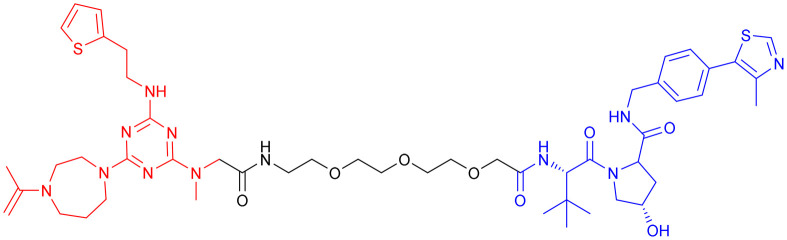	*In vitro* (HEK293-hTau and WT SH-SY5Y)	68
				*In vivo* (hTau-overexpressing mouse and 3xTg-AD mouse)	
3		CRBN	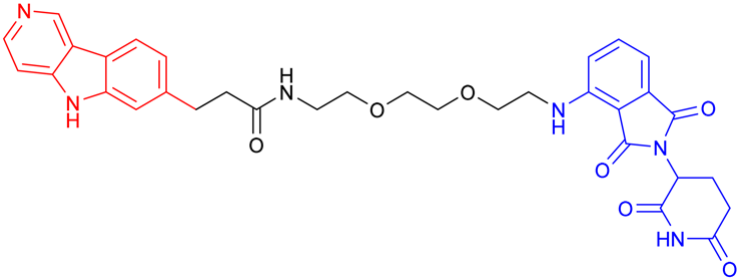	*In vitro* (FTD patient-derived neuron)	69
4			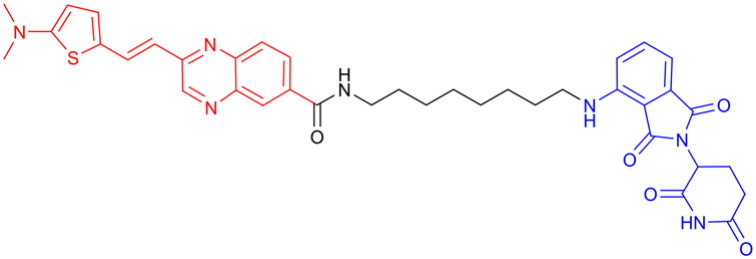	*In vitro* (HEK293-hTau)	70
				*In vivo* (hTau-overexpressing mouse)	

With CRBN serving as the recruited E3 ligase, in 2019, S. J. Haggarty *et al.* engineered an 18F-T807-based (a commonly used radioactive probe for tau in paired helical filament forms in PET brain imaging) degrader incorporating CRBN as the E3 ubiquitin ligase ligand (Table 3, PROTAC 3).^69^ This construct represents the first successful conversion of a tau PET probe into a functional degradation molecule. Using frontotemporal dementia (FTD) patient-derived neurons as a cellular model, the researchers demonstrated that 24-hour treatment with PROTAC 3 significantly reduced both total tau and p-Tau levels while showing no effect on tau in healthy control neurons. Notably, pretreatment with PROTAC 3 conferred protection against Aβ toxicity in FTD neurons, restoring neuronal survival rates to 90% and reversing stress vulnerability. However, the study presents two key limitations. First, an off-target effect was observed 4 hours post-treatment, likely arising from the E3 ligase-binding moiety, consistent with findings reported in previous research.^71^ Second, the study lacks *in vivo* validation. Even within the two patient-derived neuronal models carrying distinct tau mutations (A152T and P301L), PROTAC 3 exhibited variant-dependent degradation profiles, suggesting differences in UPS. Such variability is expected to be even more complex at the organismal level, potentially complicating translational applications.^69^

In 2024, Xie Y. *et al.* synthesized a tau-binding motif composed of a quinoxaline–thiophene hybrid structure (Table 3, PROTAC 4). In HEK293 cellular models, this compound achieved >50% degradation of both total tau and p-Tau within 12 hours at concentrations of 0.05–0.2 μM. The inclusion of a hydrophobic alkyl chain linker conferred enhanced BBB permeability, as demonstrated in hTau-overexpressing mice following intravenous administration. Treatment significantly reduced soluble and insoluble tau aggregates in the hippocampus and ameliorated cognitive deficits, although its long-term safety profile requires further investigation.^70^

In 2016, Chu *et al.* developed peptide PROTAC derived from the α- and β-tubulin peptide sequences that interact with Tau (Table 4, PROTAC 5). The PROTAC exhibited efficient cell-penetrating ability and specifically degraded endogenous tau protein. In N2a cells, SH-SY5Y cells, and primary neurons from 3xTg-AD mice, these molecules significantly downregulated tau protein levels. They also effectively mitigated Aβ-induced cytotoxicity by reducing tau levels without showing significant cellular toxicity. *In vivo* experiments further confirmed that intranasal combined with intravenous administration of this PROTAC effectively reduced tau protein levels in the cerebral cortex and hippocampal CA3 region of 3xTg-AD mice. This study was the first to report peptide-directed ubiquitin–proteasome-mediated degradation of tau protein, offering an efficient and targeted novel strategy for AD treatment.^66^ However, this PROTAC may suffer from potential issues such as low bioavailability or poor BBB penetration, complicating the administration route and increasing the difficulty of its clinical translation.

**Table 4:** Tau-targeting PROTACs(peptide)

PROTAC	POI	Structure	Cell- penetrating peptide	Efficacy validation model	Ref.
5	Tau	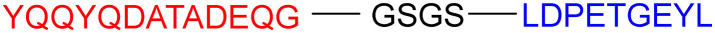	RRRRRRRR	*In vitro* (N2a cells, SH-SY5Y cells)	66
				*In vivo* (3xTg-AD mice)	
6		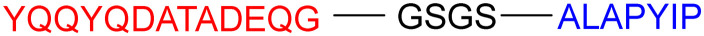	RRRRRRRR	*In vitro* (N2a cells, SH-SY5Y cells)	72

In 2018, Jiang *et al.* employed Keap1 as the E3 ubiquitin ligase in the design and development of PROTAC molecules, successfully constructing a peptide-based PROTAC targeting tau (Table 4, PROTAC 6). This PROTAC molecule efficiently degraded tau protein in neuronal cell models such as SH-SY5Y and Neuro-2a, with a maximum degradation efficiency reaching 83.92% ± 1.54%. Notably, this study was the first to introduce Keap1 into the PROTAC design system and demonstrated the potential of Keap1 as an E3 ubiquitin ligase for PROTACs in the context of AD, thereby expanding the application of PROTAC technology in the AD field.^72^

The use of peptides as targeting ligands overcomes the challenge of lacking available small-molecule ligands for certain targets. However, compared to traditional small-molecule drugs, peptides suffer from drawbacks such as high molecular weight, poor *in vivo* stability, and relatively low bioavailability. These limitations make it difficult to achieve effective therapeutic concentrations in the brain, often necessitating significantly higher doses and complicating the administration routes and pharmacokinetic profiles in animal studies. The aforementioned studies on peptide-based tau PROTACs did not delve deeply into these issues. To address these problems, future optimization of peptide-based tau degraders could consider the following directions: first, research should not be limited to evaluating protein degradation efficacy at the cellular level but should further utilize AD animal models to thoroughly investigate their BBB penetration capability and *in vivo* pharmacokinetic characteristics. Second, to enhance the metabolic stability of peptide molecules, strategies such as non-natural amino acid modifications could be employed—for example, replacing amino acids in the peptide with β-amino acids or modifying specific sites with long-chain fatty acids to increase lipophilicity and prolong half-life.^73,74^ Furthermore, innovative administration routes (*e.g.*, nose-to-brain delivery) or advanced drug delivery systems (*e.g.*, nanocomplexes) could be explored to overcome BBB limitations and improve drug distribution concentration and delivery efficiency in brain tissues.^75^

### 4.2 PROTACs targeting GSK-3β

Glycogen synthase kinase-3β (GSK-3), a serine/threonine protein kinase with phosphotransferase activity, orchestrates diverse cellular processes, including metabolism, cell cycle regulation, signal transduction, neurological function, and inflammation.^76–78^ GSK-3 exists in two isoforms, GSK-3α and GSK-3β, with both having 97% amino acid sequence identity in the catalytic domains.^79^ Among them, GSK-3β has received greater attention in the context of neurodegeneration due to its roles in tau hyperphosphorylation, Aβ plaque formation, neuroinflammation, and oxidative stress.^80–85^ Elevated levels of GSK-3β in AD patients and animal models^81,86–88^ have been linked to: first, phosphorylation of tau at serine residues, enhancing its aggregation into NFTs;^89,90^ second, astrogliosis, increased production of proinflammatory cytokines, and microglial activation, which collectively contribute to neuroinflammation;^91–94^ third, induction of BACE1 expression and modulation of γ-secretase activity, thereby promoting the amyloidogenic pathway.^90,95–97^ Given its multifaceted roles in AD-related pathologies, GSK-3β has emerged as a significant therapeutic target.

GSK-3 inhibitors can be broadly divided into two main classes: ATP-competitive and ATP-noncompetitive inhibitors.^98^ However, these inhibitors are often susceptible to the development of drug resistance, either through point mutations within the ATP-binding site that prevent inhibitor recognition or through compensatory cellular mechanisms that upregulate the target protein.^76,80^ Given that PROTAC technology can completely eliminate the target protein and thereby potentially overcome this type of drug resistance, in 2021, Xu *et al.* reported the development of the first known degrader (Table 5,PROTAC 7) targeting GSK-3β, created by conjugating a thalidomide-derived E3 ligase ligand with an ATP-competitive GSK-3β inhibitor. The compound demonstrated dose-dependent GSK-3β degradation, achieving 44.2% protein clearance at 2.8 μM in cellular models. *In vitro* neuroprotection assays revealed its ability to prevent glutamate-induced cytotoxicity in HT-22 cells, suggesting potential therapeutic effects through anti-inflammatory and cytoprotective mechanisms. However, the compound's high molecular weight raises concerns regarding bioavailability, and its pharmacological profile—including metabolic stability and toxicity—remains uncharacterized due to the absence of *in vivo* validation.^99^

**Table 5:** GSK-3β-targeting PROTACs

PROTAC	POI	E3 ligase	Structure	Efficacy validation model	Ref.
7	GSK-3β	CRBN	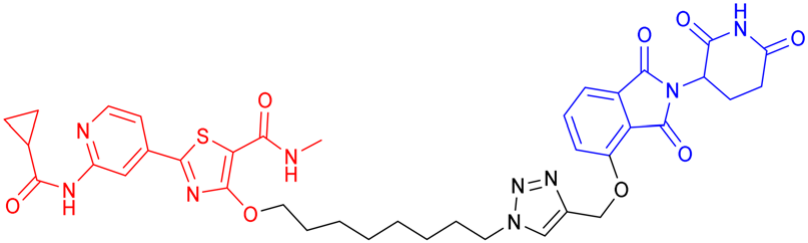	*In vitro* (PC12 cells, BV2 cells, HT-22 cells)	99
8	GSK-3α		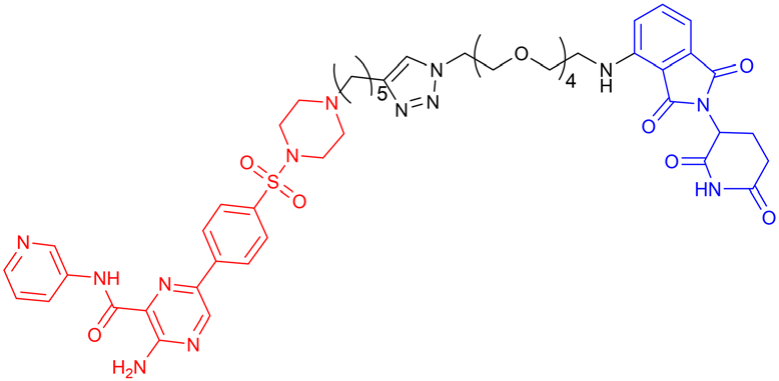	*In vitro* (Aβ oligomer-treated SH-SY5Y cells and HEK293T cells)	100
	GSK-3β			*In vivo* (okadaic acid-treated rat)	
9	GSK-3β		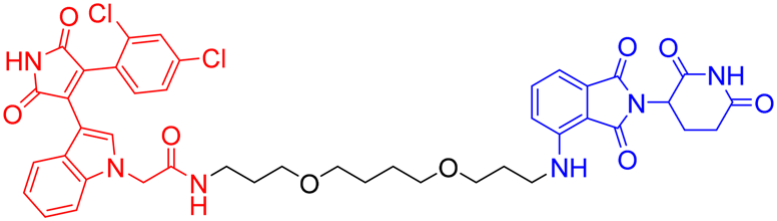	*In vitro* (SH-SY5Y cells)	101
10	GSK-3α		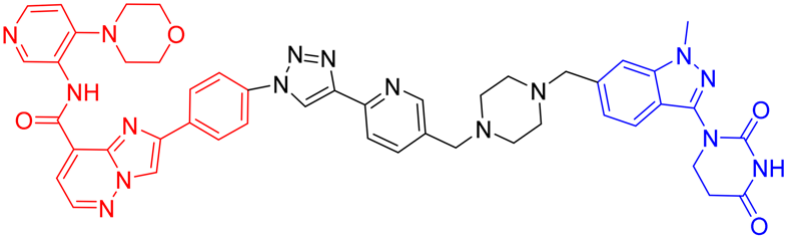	*In vitro* (HEK293 cells)	102
11	GSK-3β		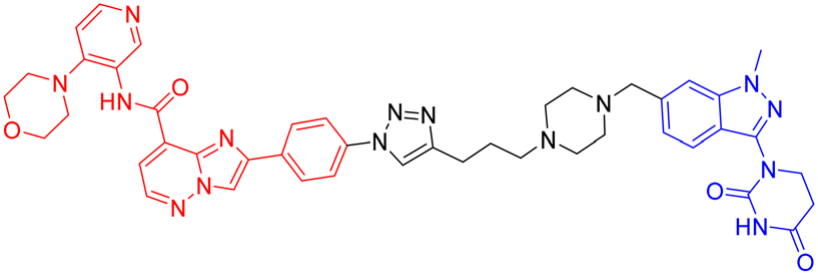		

Another study from the same year, incorporating *in vivo* experiments, further strengthened the potential of using PROTACs as GSK-3 inhibitors. Yin *et al.* employed a click chemistry platform to systematically optimize adapters, E3 ligase ligands, and GSK-3-binding moieties, ultimately identifying 3-amino-6-(4-((4-(1-(17-((2-((2,6 dioxopiperidin-3-yl)-1,3-dioxoisoindolin-4-yl)amino)-3,6,9,12,15-pentaoxaheptadecyl)-1*H*-1,2,3-triazol-4-yl)butyl)piperazine-1-yl)sulfonyl)phenyl)-*N*-pyridin-3-yl pyrazine-2-carboxamide (Table 5, PROTAC 8) as a highly selective GSK-3 degrader on an ATP-competitive manner. This compound demonstrated potent and selective GSK-3 degradation in SH-SY5Y cells, with DC_50_ values of 28.3 nM (GSK-3α) and 34.2 nM (GSK-3β), while exhibiting minimal off-target effects. PROTAC 8 effectively mitigated Aβ oligomer-induced cytotoxicity and apoptosis, demonstrating significant neuroprotective properties. *In vivo* studies revealed that intracerebroventricular administration improved okadaic acid-induced learning and memory deficits in an AD rat model, concurrently reducing hippocampal levels of both GSK-3α/β and phosphorylated tau. Notably, PROTAC 8 represents the first nanomolar-potency GSK-3 degrader with demonstrated intracerebroventricular efficacy, although its BBB permeability remained uncharacterized.^100^

In 2023, Andrea Milelli *et al.* conjugated SB-216763, an ATP-competitive inhibitor of GSK-3β, with pomalidomide to design and synthesize a series of novel PROTAC molecules. Through linker optimization, they obtained an optimal degrader, PROTAC 9 (Table 5), for targeted degradation of GSK-3β. This compound exhibited DC_50_ = 6.22 μM for GSK-3β in SH-SY5Y neuronal cells without obvious cytotoxicity. At concentrations of 0.5–1 μM, PROTAC 9 significantly reversed the neurotoxic damage induced by CuSO_4_ and Aβ_25–35_ in SH-SY5Y cells. Furthermore, using the parallel artificial membrane permeability assay (PAMPA), the effective permeability coefficient (*P*_e_) of PROTAC 9 was measured to be approximately 15.33, suggesting its potential to cross the blood–brain barrier.

In 2025, William Farnaby *et al.* employed an “orthogonal reaction linker” strategy to connect CRBN/VHL recruiters with GSK-3 binders derived from PF-367 and CMP-4732 *via* a two-step chemical reaction. From a library of 19 PROTACs, cellular screening identified two potent GSK-3 degraders (Table 5, PROTAC 10, PROTAC 11). Among them, PROTAC 10 displayed a DC_50_ of only 1.4 nM for GSK-3β degradation in HEK293 cells, with a maximal degradation efficiency reaching 93%, and selectively degraded GSK-3α and GSK-3β. Moreover, after intravenous administration in mice, the brain concentration of PROTAC 10 reached 16 nM within 2 h; at a dose of 5 mg kg^−1^, it significantly degraded GSK-3β in the mouse brain within 4 h, indicating favorable blood–brain barrier penetration. Compared with conventional PROTAC screening methods, the core advantage of this orthogonal reaction linker technology lies in its ability to rapidly and efficiently identify highly specific, highly active, and brain-penetrant targeted protein degraders, offering a novel tool for neurodegenerative disease research.^102^

### 4.3 PROTACs targeting BET proteins

Another prevalent hypothesis of AD, alongside Aβ deposition and tau protein pathology, is neuroinflammation. Similar to peripheral inflammation, the process begins with the detection of inflammatory stimuli by pattern recognition receptors (PRRs), such as Toll-like receptors (TLRs), expressed in neurons and glial cells, including astrocytes and microglia.^103^ Overactivation of these receptors has been reported in association with both Aβ aggregates and p-Tau in both *in vivo* and *in vitro* studies.^104–111^ Once activated, TLRs trigger multiple intracellular signalling cascades, notably the canonical nuclear factor-κB (NF-κB) pathway, which induces the translocation of NF-κB from the cytoplasm to the nucleus, where it binds to promoter regions of pro-inflammatory genes to initiate transcription.^112^ However, these genes are often embedded within tightly packed chromatin structures, rendering them inaccessible to NF-κB without prior chromatin remodelling. At this stage, bromodomain and extraterminal domain (BET) proteins play a critical regulatory role by “unlocking” chromatin and facilitating transcriptional activation.^113^

The BET protein family comprises four members—BRD2, BRD3, BRD4, and BRDT—all characterized by two conserved N-terminal bromodomains (BDs) 1 and 2 and a C-terminal extraterminal (ET) domain. BDs are responsible for recognizing acetylated histone residues,^114,115^ such as H3K27ac and H4K5ac, phosphorylating RNA polymerase II, and thereby linking epigenetic modifications to transcriptional activation of inflammatory genes.^116^

Among these isoforms, BRD4 is particularly noteworthy, for its dysregulation has been implicated in various conditions, including immune responses and neurological diseases.^117–119^ BRD4 directly interacts with NF-κB, facilitating the formation of chromatin–transcription factor complexes in response to inflammatory stimuli, thereby amplifying the transcription of pro-inflammatory genes.^120^ In the context of neuroinflammation, BRD4 amplifies inflammatory signalling *via* two primary mechanisms: by enhancing the transcriptional activity of NF-κB, thereby promoting the expression of pro-inflammatory cytokines like tumour necrosis factor-α and interleukin-6, or by establishing an epigenetic memory by maintaining H3K27ac enrichment in the promoter regions of inflammatory genes, leading to the overactivation of microglia and astrocytes.^116^

In addition, therapeutic interventions targeting BRD4 have been found beneficial to AD.^113,122–124^ Studies using JQ1, a selective inhibitor to the BD of BRD4, demonstrated that treatment in 3xTg-AD mice led to a reduction in pro-inflammatory mediators and decreased levels of p-Tau.^125^ Moreover, additional studies using JQ1 reported improvements in cognitive performance, attenuation of neuroinflammation, and restoration of synaptic integrity, collectively supporting the potential of BRD4 inhibition as a therapeutic strategy in AD.^126^

In 2015, James E. Bradner's team conjugated a CRBN-targeting phthalimide moiety to JQ1 through a rationally designed linker^121^ (Table 6, PROTAC 12). This compound showed the same targeting pattern as JQ1 alone, resulting in selective BET protein degradation and disruption of BRD4–transcription factor interactions. The neuroprotective potential of PROTAC 12 was demonstrated in a lipopolysaccharide (LPS)-induced microglial cell model, where it effectively reduced the expression of pro-inflammatory genes.^127^ Compared with BRD4 inhibitors, BRD4 PROTAC demonstrates superior efficacy in ameliorating pathological phenotypes, as it degrades BRD4 rather than inhibiting it.^128^

**Table 6:** BET-targeting PROTACs

PROTAC	POI	E3 ligase	Structure	Efficacy validation model	Ref.
12	BET	CRBN	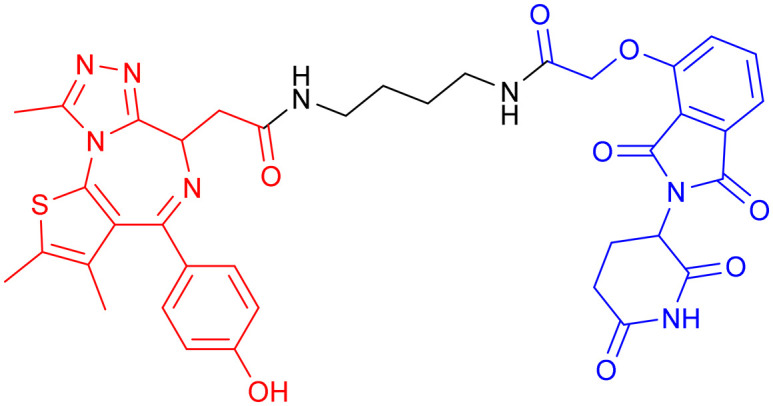	*In vitro* (WT human AML cells, LPS-treated SIM-A9 mouse microglial cells)	121

### 4.4 PROTACs targeting α-synuclein

α-Synuclein (α-Syn), a presynaptic neuronal protein, is implicated in the pathogenesis of neurodegenerative disorders, with its aberrant aggregation significantly contributing to AD core pathology.^129^ Post-mortem analyses reveal co-localization of α-Syn with Aβ and tau pathologies within the brain tissue of approximately 50% of AD patients, suggesting a synergistic pathogenic mechanism.^130,131^ α-Syn aggregation may facilitate tau hyperphosphorylation, protofibril formation, and subsequent propagation, thereby exacerbating neurodegeneration and cognitive decline in AD. Furthermore, co-aggregation of α-Syn with tau accelerates neurofibrillary tangle formation and neuronal damage.^132^ This pathological aggregation of α-Syn also initiates intracellular stress responses, including oxidative stress, mitochondrial dysfunction, and endoplasmic reticulum stress, ultimately culminating in neuronal death, thus playing a pivotal role in AD progression.^133^

In 2020, Kargbo *et al.* synthesized a series of PROTACs (Table 7, PROTAC 13–16) targeting α-Syn for AD and Parkinson's disease (PD) treatment. The PROTACs incorporated three distinct α-Syn-binding moieties (benzothiazole, indole-3-carboxamide, or phenothiazine), with each connected to E3 ubiquitin ligase ligands through either polyethylene glycol ([PEG]*n*) or cycloamine spacers. Using an ELISA-based assay in HEK293 TREX α-Syn A53T cells, PROTACs 8 and 11 demonstrated potent α-Syn degradation (>65% clearance at 1 μM), while PROTACs 15 and 16 showed intermediate efficacy (35–70% protein retention). Comparative analysis revealed that VHL-based ligands exhibited superior degradation efficiency compared to CRBN-based counterparts, while spacer length showed no significant effect on α-Syn clearance. Notably, although these compounds demonstrated encouraging *in vitro* activity, their translational potential remains limited by high molecular weights and the absence of *in vivo* efficacy data. Further optimization and preclinical evaluation are warranted to assess their therapeutic feasibility.^134^

**Table 7:** α-Syn-targeting PROTACs

PROTAC	POI	E3 ligase	Structure	Efficacy validation model	Ref.
13	α-Syn	VHL	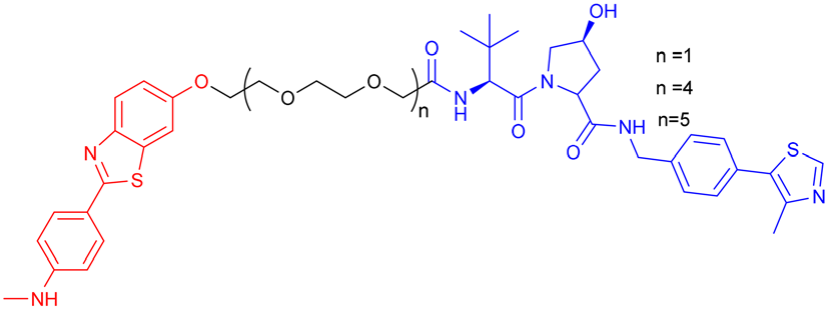	*In vitro* (HEK293 TREX α-Syn A53T cells)	134
14			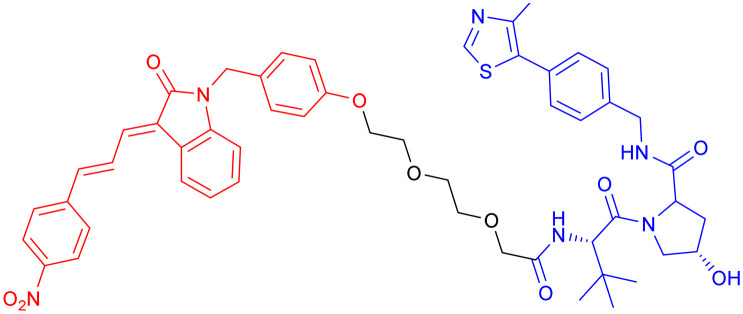		
15		CRBN	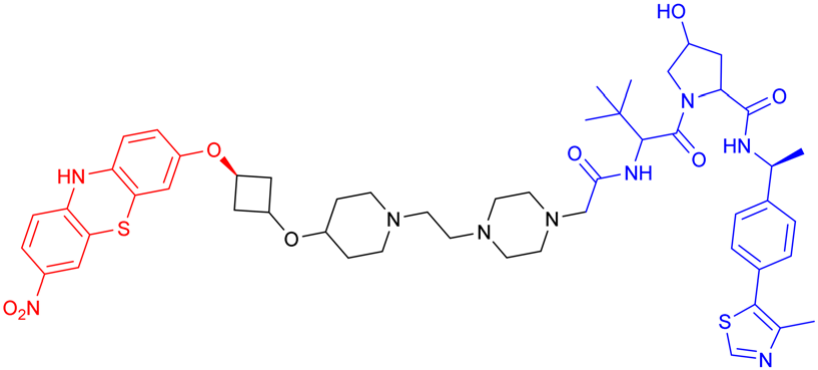		
16			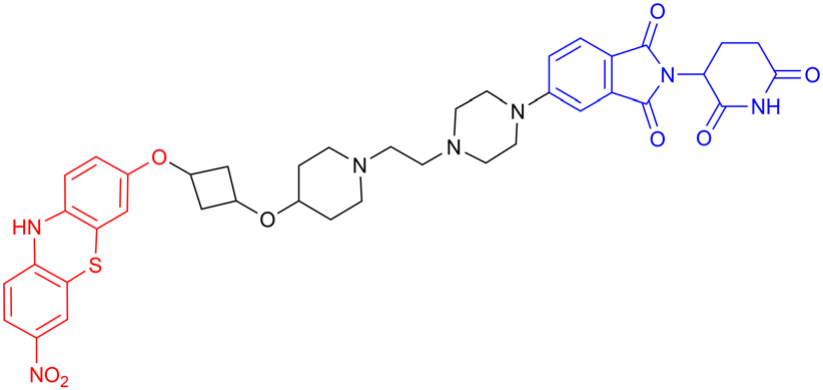		
17	Tau		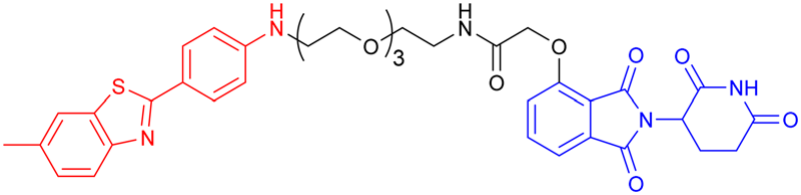	*In vitro* (SNCA OE-PFF-seeding HEK293T cells)	135
	α-Syn				
				*In vivo* (MPTP-induced PD mouse)	

Given the crosstalk between pathological proteins in neurodegeneration, a novel dual-target PROTAC (Table 7, PROTAC 17) was developed as the first degrader capable of simultaneously targeting α-Syn and tau proteins. It was designed using 2-[4′-(methylamino)phenyl]-6-methylbenzothiazole (BTA) as the warhead, chosen for its high affinity toward protein fibrils and favorable brain permeability. In a mouse model of PD induced by 1-methyl-4-phenyl-1,2,3,6-tetrahydropyridine (MPTP), 7 consecutive days of IV administration (8 mg kg^–1^ day^–1^) of PROTAC 17 resulted in significant brain exposure, with its intracerebral drug concentration sufficient to exert therapeutic effects.

Further analyses revealed that PROTAC 17 significantly reduced the accumulation of both α-Syn aggregates and p-Tau in the midbrain region while improving neuronal integrity by reducing neuronal apoptosis and synaptic damage. This bispecific degradation strategy breaks through the limitations of traditional single-target therapies in addressing complex pathological mechanisms, establishing a new framework for multi-target PROTACs in neurodegenerative diseases and informing future designs that exploit the synergistic modulation of multiple pathogenic proteins.^135^

### 4.5 PROTACs targeting P38α MAPK protein

The mitogen-activated protein kinase (MAPK) family comprises a group of serine–threonine protein kinases that can be activated by diverse extracellular stimuli, transmitting signals from the cell surface to the nucleus. p38 is a subfamily of MAPKs, and p38α has been identified as a key isoform involved in the pathological mechanisms of AD.^136^ p38α not only directly mediates the hyperphosphorylation of tau protein but also indirectly regulates tau phosphorylation by activating downstream molecules such as MAPK-activated protein kinase 2 (MK2).^137^ Furthermore, p38α participates in the generation of Aβ plaques by modulating the expression and activity of BACE1, a key enzyme in Aβ processing. Additionally, it impairs long-term potentiation (LTP), leading to synaptic loss, reduced dendritic spine density, and impaired learning and memory.^138^ Therefore, p38α represents a highly promising drug target in the field of AD research.

The benzophenone moiety of the p38α/β selective inhibitor NJK14047 induces a conformational flip of a glycine residue. Since the intrinsic conformation of phosphorylated, activated p38 (p-p38) resembles this state, p-p38 may bind this inhibitor more readily than the unphosphorylated kinase. Based on this rationale, Son *et al.* designed and synthesized PROTAC 18 (Table 8) to target the degradation of p-p38. Among 96 kinases functionally and/or structurally related to p38, PROTAC 8 selectively targeted p-p38 and significantly reduced its protein levels without affecting the phosphorylation of upstream MAPKs responsible for p38 activation. In 8- to 9-month-old 5xFAD mice and PS19 rats, intranasal administration of PROTAC 8 reduced brain levels of p-p38, decreased Aβ deposition and phosphorylated tau protein, and ameliorated AD-related pathological phenotypes and cognitive function.^139–141^ This study innovatively developed a PROTAC that directly targets a post-translationally modified (PTM) protein (p-p38) for selective degradation. It also employed intranasal delivery as a strategy to circumvent the BBB, demonstrating successful application. While aged animal models better simulate the pathological and physiological features of AD in the elderly, it is noteworthy that this study utilized only young transgenic animal models. Furthermore, the pharmacokinetic profile of the intranasal route and its potential irritation to the nasal mucosa remain to be fully elucidated. Additionally, existing evidence indicates that specifically p38α is involved in exacerbating AD pathology, while p38β is not associated with the disease. Since the p38 ligand NJK14047 used in PROTAC 18 lacks selectivity between the p38α and p38β isoforms, achieving subtype selectivity represents a potential direction for future optimization.

**Table 8:** p38α MAPK-targeting PROTAC

PROTAC	POI	E3 ligase	Structure	Efficacy validation model	Ref.
18	p38α MAPK	CRBN	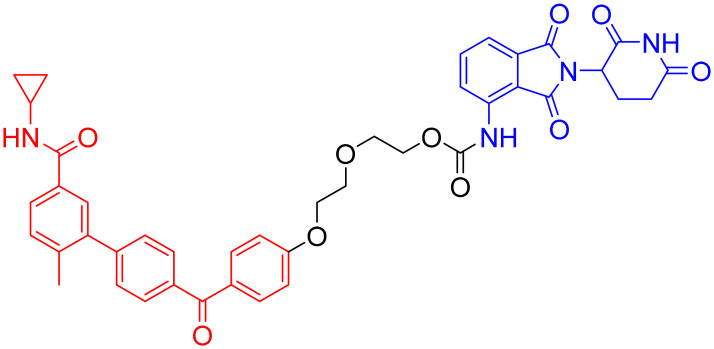	*In vitro* (C8-D1A cells, N2a cells, HT22 cells)	139

### 4.6 Other potential targets

With the advancement of PROTACs, its therapeutic applications in neurodegenerative diseases, particularly AD, continue to expand. In addition to conventional pathological targets, expanding PROTAC applications to emerging or previously understudied targets may open new therapeutic opportunities.

#### 4.6.1 ApoE4

ApoE4 is the most significant genetic driver of sporadic AD, and the core of its pathogenic mechanism stems from its unique Arg112–Cys158 salt bridge in the protein structure. ApoE4 increases the burden of neurofibrillary tangles by suppressing Aβ42 clearance and accelerating its fibrillization while simultaneously activating the sortilin-RhoA-ROCK2 pathway, which mediates the hyperphosphorylation of tau protein. Furthermore, ApoE4 inhibits ABCA1-dependent cholesterol efflux, leading to intracellular cholesterol accumulation, BACE1 activation, and elevated ceramide levels, thereby disrupting lipid homeostasis.^142^ Concurrently, it activates the NLRP3 inflammasome *via* TREM2-DAP12 signaling, promotes IL-1β secretion, and enhances astrocyte-mediated synaptic phagocytosis, ultimately resulting in reduced synaptic density. Given that ApoE4 exhibits abnormally prolonged retention in the endoplasmic reticulum, where it tends to accumulate and form stable toxic conformations, traditional small-molecule inhibitors can only partially block its downstream signaling and are unable to eliminate pathological ApoE4 protein at its source.^143,144^ Consequently, their therapeutic efficacy is limited and often accompanied by off-target effects. In contrast, PROTAC technology holds promise for achieving selective degradation of ApoE4 by targeting its Arg176 residue using ligands such as its C-terminal domain antibody fragment or AEM-28, and conjugating them with ligands for brain-enriched E3 ligases such as VHL, CRBN, or RNF114.

#### 4.6.2 Fyn kinase

Fyn kinase is a key member of the Src family of non-receptor tyrosine kinases. Compared with other Src family members, Fyn is significantly upregulated in brain regions affected by AD, and its expression level closely correlates with the severity of neurodegeneration, with its active isoform FynT being the most prominent.^145^ Fyn promotes Aβ production and tau phosphorylation by phosphorylating Tyr682 of APP and Tyr18 of tau protein, respectively, thereby disrupting axonal transport and synaptic integrity. Moreover, aberrant activation of Fyn phosphorylates the NR2B subunit of NMDA receptors, leading to intracellular calcium overload, inhibiting the brain-derived neurotrophic factor (BDNF) signaling pathway,^146^ exacerbating excitotoxicity and synaptic loss, and accelerating cognitive decline. Given the large number of Src kinase family members (such as Src, Lyn, Yes, *etc.*) with highly homologous binding pockets, existing inhibitors struggle to precisely target Fyn and often cause off-target effects, resulting in unforeseen side effects.^147^ In contrast, PROTAC technology relies only on E3 ligases that are specifically or highly expressed in target cells (*e.g.*, neurons). Even if the Fyn-targeting ligand exhibits cross-binding to homologous kinases, PROTACs can achieve selective degradation of Fyn and simultaneously overcome the drug resistance issues associated with traditional inhibitors. Therefore, building on existing inhibitor research, developing PROTACs based on brain-specific or highly expressed E3 ligases holds promise for achieving selective degradation of Fyn protein in the nervous system, thereby offering a more targeted and disease-modifying therapeutic strategy for AD treatment.

#### 4.6.3 Hypoxia/HIF1α signaling

Cerebral hypoxia is closely linked to the onset and progression of neurodegenerative diseases such as AD. The central nervous system is highly sensitive to oxygen supply, and hypoxia can promote the deposition of amyloid plaques. In the brain tissue of AD patients under hypoxic conditions, multiple signaling pathways are aberrantly activated, further exacerbating oxidative stress.^148,149^ Under hypoxia, HIF-1α accumulates in neurons and can trigger the transcriptional expression of vascular endothelial growth factor (VEGF) and BACE1, thereby increasing Aβ deposition and neuritic plaque formation.^150,151^ Simultaneously, HIF-1α regulates multiple signaling pathways including the mTOR pathway, some of which are involved in mediating abnormal phosphorylation of tau protein. Given its key regulatory role in AD pathology, HIF-1α has emerged as a potential therapeutic target of considerable interest.

However, because the binding interface of HIF-1α is typically broad and shallow, lacking a well-defined small-molecule binding “pocket,” direct targeting for drug design has proven highly challenging.^152^ Current HIF-1α inhibitors in clinical stages, such as EZN-2968 and BAY 87-2243, mostly function by indirectly modulating its upstream synthesis and degradation pathways or affecting its chaperone proteins, resulting in limited targeting specificity.^149^ In the future, it may be possible to draw inspiration from the binding mode of HIF-1α with endogenous proteins such as HSP90 to design peptide-based ligands for HIF-1α. By applying PROTAC technology, direct targeted degradation of HIF-1α could be achieved, thereby offering a new strategic direction for the treatment of AD.

#### 4.6.4 PI3K/AKT/mTOR pathway

Dysregulation of the PI3K/AKT/mTOR pathway plays a significant role in the pathological progression of AD. This pathway is not only involved in Aβ-induced oxidative stress and aberrant tau phosphorylation but also exacerbates the formation of neurofibrillary tangles by regulating the activity of downstream kinases such as GSK-3.^153,154^ Consequently, targeting this pathway has emerged as a potential therapeutic strategy for AD. Notably, PROTAC molecules directed against key nodes of this pathway, such as PI3K and AKT, have been successfully developed and have demonstrated advantages in overcoming resistance to conventional inhibitors, providing a proof of concept for AD treatment based on protein degradation technology.^155,156^

Within this pathway, mTOR serves as a critical regulatory hub. It promotes tau phosphorylation by influencing multiple kinases, including GSK-3 and CDK5, and its hyperactivation contributes to tau accumulation and synaptic dysfunction.^157,158^ Although the mTOR-specific inhibitor rapamycin has shown some neuroprotective effects in preclinical studies, its long-term application is often limited by issues such as immunosuppression, nephrotoxicity, and drug resistance.^159^ Therefore, building on existing inhibitors, developing mTOR-targeted PROTAC degraders holds promise for achieving more sustained and precise intervention, offering a novel therapeutic solution for AD.

#### 4.6.5 HDAC11

Histone deacetylases (HDACs) represent a crucial class of epigenetic regulatory enzymes.^160^ Among them, HDAC11 has been identified in recent years as a member of the Class IV HDAC family. Studies have revealed that HDAC11 expression is significantly upregulated in the brains of AD patients. Its expression level shows a positive correlation with the number and area of Aβ plaques. Furthermore, regions with high HDAC11 expression exhibit markedly increased activation of microglia, indicating a close association between HDAC11 and the characteristic amyloid plaque deposition as well as neuroinflammatory markers in AD. In the 5xFAD transgenic mouse model, pharmacological inhibition of HDAC11 activity enhances the phagocytic clearance of Aβ protein by microglial cells (such as the BV2 cell line), alleviates neuroinflammation, and ultimately improves cognitive function in mice.^161^

However, the HDAC family comprises 11 members with highly similar catalytic pocket structures, making it difficult for inhibitors to avoid affecting other subtypes (*e.g.*, HDAC6) and leading to off-target side effects. Therefore, compared to developing HDAC11 inhibitors, PROTAC molecules designed for the direct degradation of HDAC11 not only offer higher subtype selectivity but also advance from “functional inhibition” to “target elimination.” This approach holds promise for more thoroughly blocking all pathogenic functions of HDAC11, thereby providing a therapeutic strategy with greater selectivity and longer-lasting efficacy.

## 5. Challenges and future directions for PROTAC-based AD drugs

### 5.1 BBB penetration and its implications for PROTAC design

The BBB is created by a special system of brain endothelial cells (BECs), pericytes (PCs), the capillary basement membrane, and the terminal branches (“end-feet”) of astrocytes (ACs). The key function of the BBB is to safeguard the CNS from potentially harmful/toxic substances circulating in the bloodstream by tightly controlling the entry of cells and molecules, including nutrients, metabolites, and components of the immune system, into the brain parenchyma.^162^

Although small molecules such as PROTACs are generally considered to possess better BBB permeability compared to biomacromolecules, their actual permeability varies from molecule to molecule. For instance, PROTACs 3, 7, and 13–16 have exhibited promising degradation efficiency in *in vitro* cellular assays; however, due to their relatively high molecular weights, their BBB penetration is likely poor, which hinders their ability to exert targeted effects *in vivo*. Consequently, subsequent *in vivo* validation was not conducted, and related research remained at the cellular level. Although some PROTAC molecules, such as PROTAC 2, PROTAC 4, and PROTAC 8, have been shown in preclinical models to cross the BBB *via* passive diffusion or active transport, their delivery efficiency is still constrained by efflux transporter systems. Among these, active efflux mediated by transporters such as P-glycoprotein (P-gp) and breast cancer resistance protein (BCRP) represents a major limiting factor, which can reduce the brain-to-plasma exposure ratio of their substrate drugs by threefold. Consequently, achieving effective therapeutic concentrations in the brain still necessitates administration routes with suboptimal clinical compliance, such as intracerebroventricular injection or combination therapies. Overcoming drug efflux mediated by efflux transporters has thus become a major challenge in central nervous system drug delivery.

In comparison to antibodies, which are large, integrated molecules with limited BBB permeability, PROTACs offer structural flexibility, allowing for optimization through rational chemical design or *in silico* prediction models.^163^ Key parameters influencing PK in CNS include hydrogen-bond donor (HBD) count and topological polar surface area (TPSA).^164^ Reducing both HBD and TPSA is generally associated with improved brain exposure and reduced efflux ratio. Nevertheless, such modifications must be carefully balanced, as lower TPSA may also lead to increased hepatic clearance. Insights from a study developing small-molecule inhibitors of leucine-rich repeat kinase 2 (LRRK2) for PD highlight this trade-off between brain penetration and systemic metabolism.^165^

In addition to passive permeability, certain endogenous transporters expressed on the apical side of BECs may facilitate CNS delivery. For example, the proton-coupled organic cation (H^+^/OC) antiporter recognizes nitrogen-containing moieties^166^—a property that has informed the design of transporter-conscious CNS drugs such as antihistamines (*e.g.*, diphenhydramine, chlorpheniramine) and some of the first generation of Alzheimer's therapeutics (*e.g.*, memantine, donepezil).

Another promising delivery strategy is by utilizing the receptor-mediated transcytosis, which can actively transport therapeutic molecules across the BBB. This mechanism has inspired the development of PROTAC–antibody conjugates (PACs), analogous to ADCs mainly explored in oncology. In proof-of-concept studies, anti-HER2 antibodies have been conjugated to PROTACs *via* VHL ligands to promote intracellular degradation of proliferative targets.^167^ Following receptor-mediated endocytosis, the antibody and linker are cleaved in the endosome, releasing the PROTAC into the cytoplasm.^166^ While these studies have primarily focused on tumour targeting rather than BBB transport, lessons can be drawn from transferrin receptor-targeted ADCs—where anti-transferrin fragments are fused to the Fc domain to enhance brain delivery^42^—suggesting potential applicability of similar strategies for CNS-targeted PACs.

Finally, the physicochemical characteristics of PROTACs, including molecular weight, ionization state, lipophilicity, and formulation, collectively influence BBB penetration. Unlike large biologics such as antibodies, PROTACs benefit from modularity and tunability, making the design of BBB-permeable candidates both more flexible and more achievable with rational medicinal chemistry approaches.

### 5.2 Degradation challenges and opportunities

The protein degradation specificity of PROTAC technology remains limited by multiple challenges. First, the target protein requires a suitable ligand. For target proteins lacking well-defined small-molecule ligands, such as tau, peptide-based ligands can be employed for linkage, as exemplified by PROTACs 5 and 6. For targets with established small-molecule inhibitors, such as GSK-3β, PROTACs derived from different inhibitors, such as PROTACs 7–9, may exhibit varied degradation efficiencies. Therefore, it is necessary to screen and optimize the most appropriate ligand. Second, the choice of linker can influence the properties of PROTACs. Research by Andrea Milelli and colleagues demonstrated that linkers of different lengths can significantly affect degradation activity. However, in other cases, such as PROTACs 13–16, the linker showed no obvious impact on degradation activity. This suggests that the role of the linker may vary depending on the target and requires evaluation within specific systems. Finally, currently available E3 ubiquitin ligase ligands for PROTAC design remain limited. Although the human genome encodes more than 600 E3 ubiquitin ligases, practical applications still rely heavily on a few types, particularly CRBN and VHL. This limitation restricts the range of targetable proteins and may lead to off-target effects, as illustrated by the off-target phenomenon observed with PROTAC 3. A key underlying reason for this limitation lies in the intrinsic characteristics of E3 ligases.^168^ First, E3 ligase expression levels vary substantially across tissues. The ubiquitous distribution of commonly employed ligases such as CRBN and VHL can increase the risk of off-target degradation, thereby complicating tissue-specific therapeutic interventions. Second, many E3 ligases lack well-defined binding pockets amenable to small-molecule engagement. Their relatively flat or conformationally flexible protein surfaces pose major challenges to the discovery of high-affinity ligands, contributing to the scarcity of “drug-ready” E3 ligases.^169^ Consequently, the development of novel E3 ligase ligands represents a crucial step toward enhancing both the selectivity and the applicability of PROTAC technology.^170^ To accelerate the discovery of novel E3 ubiquitin ligases in PROTAC development, artificial intelligence (AI)-driven tertiary structure prediction tools, such as AlphaFold and RoseTTAFold, have emerged as promising platforms. The high-resolution structural models of the full proteome released by AlphaFold include a large number of three-dimensional conformational data of E3 ubiquitin ligases that had not been previously resolved. Leveraging the predicted structures of E3 ligases, researchers can efficiently screen potential E3 ligands through techniques such as virtual screening, molecular docking, and pharmacophore modeling, or design small-molecule ligands *de novo*. These advances make it possible to rationally design PROTACs targeting previously difficult-to-target ligases and substrates, thereby expanding the ligase toolbox and improving the precision of targeted protein degradation.^171^

Abnormal UPS functioning has also been reported in patients with neurodegenerative diseases, including AD, PD and HD.^172–174^ In AD, both soluble and insoluble Aβ species have been shown to disrupt UPS activity by inhibiting proteolytic signalling.^175,176^ Tau aggregates, however, exhibit a more complex relationship with the UPS, with evidence suggesting both upregulation and downregulation of tau-targeting E3 ligases depending on the disease stage and brain region.^177–179^ These findings highlight that an impaired UPS should be taken into consideration when characterizing and optimizing PROTAC candidates for neurodegenerative conditions.

### 5.3 Hook effect and the emergence of drug resistance

In practical applications, PROTAC technology faces two major challenges: the “hook effect” and drug resistance. The “hook effect” describes a phenomenon where, beyond a certain concentration range, the degradation efficiency of the target protein paradoxically decreases as the PROTAC concentration increases. This occurs because excess PROTAC molecules tend to preferentially form inactive binary complexes with either the E3 ligase or the target protein, competitively inhibiting the assembly of the functional ternary complex. This phenomenon is widely observed in various PROTACs (*e.g.*, PROTAC2) and presents unique challenges for defining the therapeutic window and dosage regimen. To overcome this effect, current optimization strategies focus primarily on molecular design, including modulating linker length and rigidity to stabilize the ternary complex; modifying the warhead structure to reduce the propensity for binary binding;^180^ designing molecules capable of recruiting two distinct E3 ligases to minimize the accumulation of non-productive complexes;^181^ and constructing multivalent nano-degradation chimeras to promote stable ternary complex formation.^182^

Furthermore, PROTACs can potentially trigger adaptive drug resistance. Research indicates that prolonged exposure of cells to PROTACs reliant on particular E3 ligases like VHL or CRBN may lead to functional impairment due to genetic changes in the fundamental elements of the E3 ubiquitin ligase complex.^183^ Consequently, this could diminish the effectiveness of recruiting and degrading PROTACs. Therefore, in chronic conditions like AD that demand extended therapeutic interventions, it is crucial to proactively address and devise approaches to mitigate analogous drug resistance challenges.

## 6. Conclusions

Since its first report in 2001, PROTAC technology has demonstrated considerable potential as a targeted protein degradation strategy. Over the past two decades, a growing body of research suggests that PROTACs with protein-degrading functions hold promise as novel therapeutic approaches for neurodegenerative diseases. However, the relatively large molecular weight of PROTAC molecules presents significant challenges for BBB penetration; meanwhile, the limited availability of E3 ubiquitin ligases in brain tissues also remains a key obstacle in the current development of PROTAC-based therapies for AD. To date, no PROTAC molecule has entered clinical trials in the AD field, and systematic evaluations of the safety, efficacy, and pharmacokinetic properties of relevant PROTACs in humans are still lacking.

Moving forward, PROTAC drug development for neurodegenerative diseases such as AD should focus on two major directions: first, the development of efficient brain-targeting strategies for PROTAC molecules to overcome the key hurdles in clinical translation. Currently, how to increase effective brain exposure of PROTACs represents the central challenge for clearing pathological protein aggregates such as Aβ and tau. To this end, researchers are actively exploring various brain-targeting technologies. For instance, Roche modified an Aβ antibody with transferrin receptor binding, which increased its efficiency in clearing cerebral Aβ by threefold while reducing the required dose to one-fifth of the original.^184^ In another study, lipid nanoparticles (LNPs) were used to encapsulate a tau-targeting PROTAC and delivered across the BBB *via* transferrin receptor-mediated transport, demonstrating enhanced tau clearance activity and superior BBB penetration.^185^ Such strategies that precisely integrate BBB-penetrating enhancer modules (*e.g.*, transferrin receptor binders) with the core PROTAC structure can significantly improve brain accumulation efficiency while maintaining degradation activity. This offers a feasible technical route for designing brain-targeted PROTACs and holds promise for fundamentally addressing the core issue of insufficient brain exposure and ineffective clearance of pathological protein aggregates like Aβ and tau. Second, AI-driven compound structure optimization strategies can be leveraged to enhance the development efficiency of PROTAC drugs. In the rational design of PROTAC molecules, various AI models have enabled full-process support from linker generation to molecular optimization. For example, the diffusion model DiffPROTACs developed by a team at ShanghaiTech University can accurately generate PROTAC linkers based on given ligands; after fine-tuning on a PROTAC dataset, the generated PROTAC molecules achieved a validity rate of 93.86%, providing an efficient tool for linker optimization in AD-related PROTACs.^186^ Other AI technologies screen candidate PROTACs by simulating ternary complex conformations of POI-PROTAC-E3 ligase and evaluating metrics such as binding RMSD and binding free energy, followed by validation through molecular dynamics (MD) and free energy perturbation (FEP) simulations, thereby yielding PROTAC candidates with superior affinity compared to traditional methods.^187^ Furthermore, studies show that AI can be applied throughout the entire PROTAC design process. For instance, the team at Insilco Medicine first generated a PKMYT1 inhibitor using the Chemistry42 platform, then optimized the core structure of a PROTAC molecule based on it, ultimately obtaining a bifunctional PROTAC that achieved 80–90% degradation efficiency in experiments and exhibited favorable pharmacokinetic properties.^188^ Applying these AI technologies directly to PROTAC drug development can substantially shorten R&D cycles, increase success rates, and accelerate the translation and application of PROTAC technology in neurodegenerative diseases such as AD.

Compared with small-molecule inhibitors used clinically for various complex neurological disorders, PROTAC technology possesses unique advantages. In the future, with continued breakthroughs in drug delivery strategies and AI-driven drug design, PROTACs are expected to bring new hope for the treatment of AD.

## Conflicts of interest

There are no conflicts to declare.

## Data Availability

No primary research results, software or code have been included and no new data were generated or analysed as part of this review.
